# Twist2 Is Upregulated in Early Stages of Repair Following Acute Kidney Injury

**DOI:** 10.3390/ijms18020368

**Published:** 2017-02-10

**Authors:** Elizabeth A. Grunz-Borgmann, LaNita A. Nichols, Xinhui Wang, Alan R. Parrish

**Affiliations:** Department of Medical Pharmacology and Physiology, School of Medicine, University of Missouri, Columbia, MO 65212, USA; grunze@health.missouri.edu (E.A.G.-B.); nicholsla@health.missouri.edu (L.A.N.); wangxinhui89@gmail.com (X.W.)

**Keywords:** acute kidney injury, aging, dysrepair, *Kim-1*, mercuric chloride, *Twist2*

## Abstract

The aging kidney is a marked by a number of structural and functional changes, including an increased susceptibility to acute kidney injury (AKI). Previous studies from our laboratory have shown that aging male Fischer 344 rats (24 month) are more susceptible to apoptosis-mediated injury than young counterparts. In the current studies, we examined the initial injury and early recovery phases of mercuric chloride-induced AKI. Interestingly, the aging kidney had decreased serum creatinine compared to young controls 1 day following mercuric chloride injury, but by day 4, serum creatinine was significantly elevated, suggesting that the aging kidney did not recover from injury. This conclusion is supported by the findings that serum creatinine and kidney injury molecule-1 (*Kim-1*) gene expression remain elevated compared to young controls at 10 days post-injury. To begin to elucidate mechanism(s) underlying dysrepair in the aging kidney, we examined the expression of Twist2, a helix-loop-helix transcription factor that may mediate renal fibrosis. Interestingly, *Twist2* gene expression was elevated following injury in both young and aged rats, and Twist2 protein expression is elevated by mercuric chloride in vitro.

## 1. Introduction

A number of studies have linked aging with a higher risk for acute kidney injury (AKI) [1,2]. The incidence of AKI is 3.5 times higher in patients over 70 than those under 70, while patients older than 80 years are 5.0 times more likely to develop AKI [3]. Other studies have shown that elderly patients (≥65 years) have 10 times the incidence rate of AKI [4]. Animal studies recapitulate the increased susceptibility of the aging kidney to injury. There is an age-related increase in acetaminophen nephrotoxicity in male Fischer 344 rats [5]. Miura et al. [6] demonstrated that renal slices of old rats were more susceptible to anoxia than slices from young counterparts. Previous studies from our laboratory showed similar results; renal slices from aged Fischer 344 rats fed ad libitum, but not aged caloric-restricted animals which do not develop renal fibrosis and dysfunction [7], were more susceptible to ischemic injury when compared with slices from young animals as assessed by histological and biochemical evaluation [8]. These ex vivo studies demonstrated that the aged proximal tubular epithelial cells had an inherent susceptibility to injury.

Elderly patients are less likely to recover from AKI; the percentage of elderly patients who did not recover renal function was 31.3% compared with 26% of younger cohorts [9]. Hospitalized AKI patients requiring dialysis are older than their counterparts not requiring dialysis (63.4 vs. 47.6 years) [10]. Mortality following AKI may also be increased in the elderly [11,12]. Recovery from AKI, as determined by time to normalization of serum creatinine, was three-times as long in elderly (mean 67.1) compared to young (32.3) patients (32 vs. 11.4 days, respectively) [13]. A higher percentage of surviving elderly (>65 years) patients did not recover renal function as compared to younger patients in an analysis of 17 studies [14]. Fortunately, data from animal studies are in agreement with the clinical findings. Zinc-α(2)-glycoprotein (Zag), an inhibitor of epithelial cell proliferation, is elevated (6.4-fold) in proximal tubular epithelial cells from aged mice and is responsible, in part, for decreased repair following acute injury [15].

There are a number of animal models of toxicant-induced AKI, including gentamicin, glyercol, and cisplatin [16]; Mercuric chloride-induced AKI in laboratory animals has been used to elucidate mechanisms underlying AKI [17,18]; early markers of injury include a significant loss of the brush border enzymes alkaline phosphatase and g-glutamyltranspeptidase in the urine [19,20]. Mercuric chloride-induced AKI is a relatively pure nephrotoxic model; injury is not responsive to anti-inflammatory treatment [21,22]; this allows the investigation of injury and repair without the important, yet confounding, variable of inflammation-mediated changes.

## 2. Results

**Figure 1:**
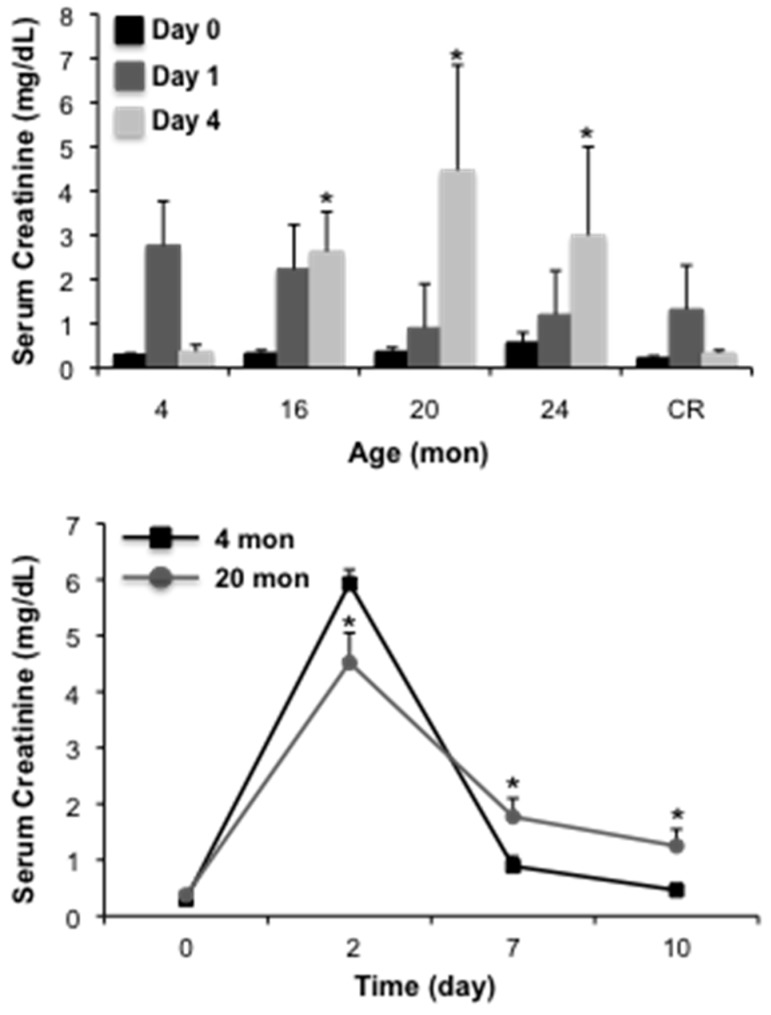
The impact of aging on mercuric chloride-induced acute kidney injury (AKI). Rats were challenged with 2 mg/kg mercuric chloride and AKI was assessed by serum creatinine. In the **top** panel, rats at different ages were challenged and harvested 1 or 4 days following challenge; In the **bottom** panel, 20 month (mon) old rats were challenged with mercuric chloride and serum creatinine was assessed 2, 7 or 10 days following injury. Each data point represents the mean + SEM of 3–4 animals, * indicates a significant difference from young control (4 mon).

**Figure 2:**
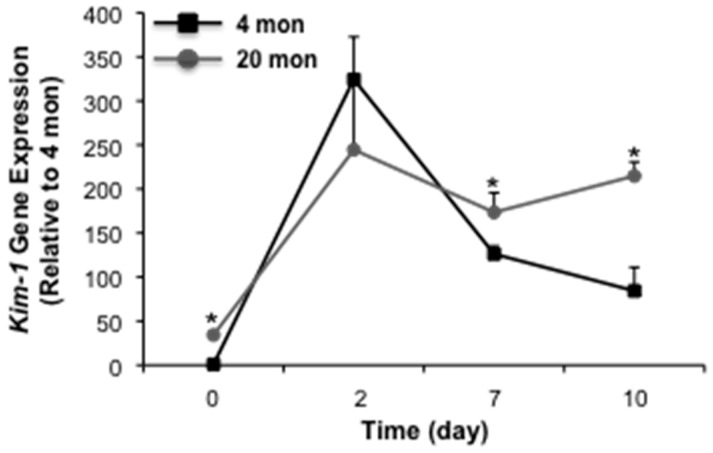
The impact of aging on mercuric chloride-induced *Kim-1* gene expression. Twenty mon old rats were challenged with mercuric chloride and *Kim-1* gene expression was assessed 2, 7 or 10 day following injury. Each data point represents the mean + SEM of 3–4 animals, * indicates a significant difference from young control (4 mon).

Previous studies from our laboratory have demonstrated that aged male Fischer 344 rats are more susceptible to cisplatin-induced AKI than young counterparts [23], an effect that is linked to an increased susceptibility to apoptosis [23,24]. Given the complexity of cisplatin-induced injury, which involves cellular injury due to necrosis and apoptosis, as well as inflammation [25,26], we wanted to determine the impact of aging strictly on proximal tubular epithelium injury in AKI. In the first set of studies, we challenged male Fischer 344 rats with 2 mg/kg mercuric chloride and assessed injury by measuring serum creatinine levels at 1 or 4 days post-injury. Interestingly, serum creatinine levels were not different from young (4 month) controls in 16, 20 or 24 month animals at day 1 (Figure 1). However, at day 4, serum creatinine levels remained elevated in the aged (16–24 month), but not young rats. The injury profile of rats subjected to life-long caloric restriction, which attenuates age-dependent renal dysfunction [7], was similar to young controls (Figure 1). A similar pattern of results was seen in a follow-up study with an extended time course; in these studies we used 20 month rats to characterize an injury/repair model that will allow for an extended time course. At day 2 following injury, serum creatinine was elevated in both young (4 month) and aged (20 month) rats, however, levels were significantly higher in young animals (Figure 1). At day 7 and 10 following injury, serum creatinine was elevated in aged animals relative to young controls. Importantly, at day 10, serum creatinine remained elevated in aged animals, 3.37-fold relative to control. We also examined gene expression of *Kim-1*, a marker of AKI [27,28] and potential mediator of renal fibrosis in chronic kidney disease (CKD) [29]. Consistent with our previous studies [8,30,31], *Kim-1* was elevated in aged rats relative to young controls (Figure 2). Mercuric chloride-induced AKI caused a substantial elevation in *Kim-1* in both young and aged rats. In young rats, *Kim-1* gene expression declined, although not back to control levels, over the 10-day time course. In the aged rats, however, *Kim-1* gene expression remained elevated at day 7 and 10 post-injury (Figure 2). Taken together, these data suggest that the aging kidney does not recover from acute kidney injury relative to young controls.

**Figure 3:**
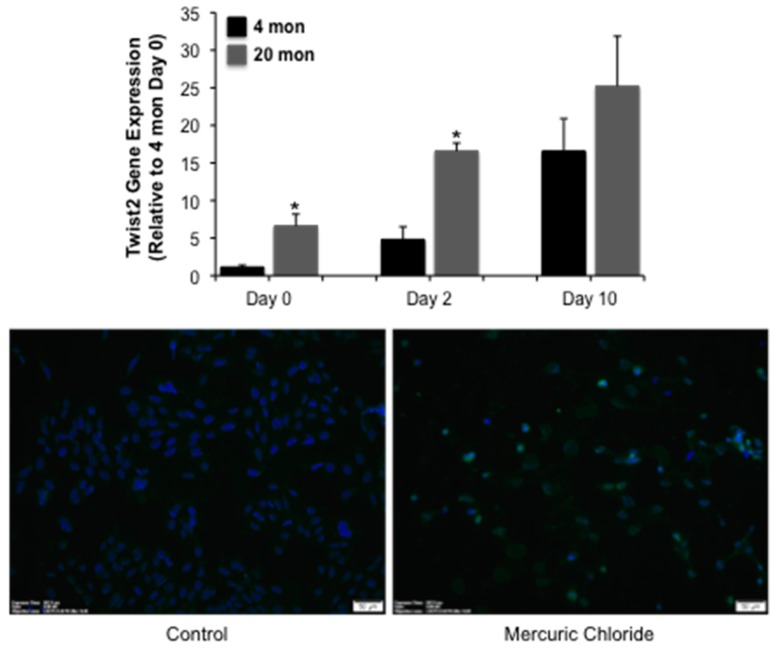
The impact of mercuric chloride on Twist2 expression. In the top panel, 20 mon old rats were challenged with mercuric chloride and *Twist2* expression was assessed 2 or 10 day after injury. Each data point represents the mean + SEM of 3–4 animals, * indicates a significant difference from young control (4 mon). In the bottom panel, DCT209 cells were challenged with 5 µM mercuric chloride for 24 h and Twist2 expression assessed by immunofluorescence (green staining), scale bar = 50 µm; similar results were seen in two replicate experiments.

We next examined several molecular endpoints to begin to identify putative mediators of dysrepair in the aging kidney. *Twist2* gene expression is increased in aged animals relative to young controls (Figure 3A). Interestingly, expression is elevated in both young and aged animals following mercuric chloride challenge at day 2, and remains elevated at day 10; a significant difference between young and aged animals is seen at day 0 and 2, but not day 10 (Figure 3A). This suggests that Twist2 may be required for repair following AKI. The finding that Twist2 staining is increased in DCT209 cells following a 24 h challenge with 5 µM mercuric chloride (Figure 3B) supports the hypothesis that Twist2 is upregulated by injury. Taken together, these data suggest that Twist2 is a novel pathway activated in AKI.

## 3. Discussion

Previous studies from our laboratory [8,23,24] and others [5,6,15] have shown that the aging kidney has an increased susceptibility to acute injury, which parallels the increased incidence of AKI in the elderly [1,2,3,4]. The data presented in this paper suggest, however, that this may be dependent on the form of injury. Our data demonstrate that initial mercuric chloride-induced injury (days 1 and 2) is not increased in the aging kidney, but renal dysfunction is elevated in the early stages of repair (days 4–10). Importantly, this data suggests that the mercuric chloride-induced AKI model allows for the study of repair without the confounding variable of increased injury that has been reported in aging rats for other nephrotoxicants.

Maladaptive repair in the kidney following AKI may lead to fibrosis and the exacerbation of CKD [32]. Renal repair is complex process that involves dedifferentiation of surviving tubular epithelium, proliferation and migration and, ultimately, redifferentiation into functional tubular epithelium [33,34]. Previous studies have shown that decreased proliferation is a critical determinant of AKI in the aged mouse kidney [15].

We have shown that the aging male Fischer 344 rat is characterized by a loss of N-cadherin, the predominant cell-cell adhesion molecule in the proximal tubular epithelium of the rat and humans [35], and α-catenin [36]. Knockdown of α-catenin in cells results in increased susceptibility to apoptosis, but not necrosis [23,24] consistent with the increased susceptibility of the aging kidney to cisplatin [23], but not mercuric chloride-induced AKI. Knockdown of α-catenin is not associated with decreased proliferation, but alterations in migration [37,38]. Given the important role of the cadherin-catenin complex in epithelial differentiation, we hypothesize that the aging kidney may not be able to completely re-differentiate following AKI. This is consistent with the recently elucidated role of partial epithelial-to-mesenchymal transition (pEMT) in the development of fibrosis and CKD [39,40].

Twist2 regulates gene expression via the 5′-nCAnnTGn-3′ consensus sequence [41] and has a high degree of homology (66% identical) and an overlapping pattern of cellular expression with Twist1 [42]. The expression of Twist1, however, was not changed during the mercuric chloride-induced injury in either young or aged rats (data not shown). Twist2 inhibits osteoblast and myoblast differentiation [42,43]; in terms of pathophysiology, Twist2 drives epithelial-to-mesenchymal transition in tumors [44,45,46]. We have identified overexpression of Twist2 as a potential mediator of fibrosis in the aging kidney (data not shown). Given the role of Twist2 in EMT, we hypothesized that elevated Twist2 may accompany dysrepair due to a failure to of tubular epithelium to redifferentatiate in the aging kidney. While Twist2 is elevated during the injury phase in aged rats, unexpectedly, in both young and aged rats Twist2 expression is increased during the early repair phase, suggesting that it has a role in repair. Twist2 expression is also elevated following cisplatin-induced AKI (data not shown), supporting this conclusion. Future work will focus on determining if both Kim-1 and Twist2 expression remain elevated compared to young controls over an extended time course of repair in the mercuric chloride-induced AKI model, and if these changes are associated with accelerating the development of renal fibrosis and dysfunction in the aged animals.

## 4. Methods

### 4.1. Animals

Male Fisher 344 rats were obtained from the NIA colony. Animals received a single intraperitoneal (IP) injection of 2 mg/kg mercuric chloride. On the day of the experiments, rats were anesthetized with ketamine (80–120 mg/kg)/xylazine (5–10 mg/kg) via IP injection; a cardiac puncture was performed to obtain blood. Serum creatinine was analyzed using validated commercially available assays (Beckman-Coulter, Brea, CA, USA) on an automated clinical chemistry analyzer (Beckman-Coulter AU680, Brea, CA, USA) in the Comparative Clinical Pathology Services, LLC laboratory at the University of Missouri. All animal experiments were approved (AUP 6752 approved 4/4/2011, renewed 2/12/2013) by the Animal Care and Use Committee at the University of Missouri in accordance with the NIH.

### 4.2. Gene Expression

RNA was isolated from 20 to 50 mg kidney tissue with the RNeasy mini kit (Qiagen cat#74104, Germantown, MD, USA).

cDNA was generated from 2 µg RNA using the High Capacity cDNA Synthesis Kit (ABI Enzyme cat# 4368814, Foster City, CA, USA). Quantitative PCR was performed in duplicate with 50 ng cDNA/reaction using Taqman assay with TaqMan Fast Advanced Master Mix (Applied Biosystems cat #4444557, Foster City, CA, USA) and the ABI7900HT instrument. The cycling conditions were 95 °C for 20 s, 95 °C for 1 s, 60 °C for 20 s. Steps 2 and 3 were then repeated 40 times. Relative quantitation was performed using the Pfaffl method [47] normalized to Casc3. Kim-1: Assay ID: Fn00597703_m1; Twist2: Assay ID Rn00572482_m1.

### 4.3. Immunofluorescence

DCT209 cells were plated into 2-well cell culture treated chamber slides and allowed to grow to approximately 90% confluence. Cells were then treated with 5 mM mercuric chloride for 24 h. Following treatment, cells were washed with 1X Dulbecco’s phosphate-buffered saline and fixed in 2% paraformaldehyde for 10 min. Cells were then washed with 1X phosphate-buffered saline (PBS) for 10 min twice, followed by a 10 min incubation in a 1% solution of Triton X-100 for permeabilization. Cells were then blocked for 1 h in a 1% bovine serum albumin solution followed by an overnight incubation at 4 °C in Twist2 primary antibody (LifeSpan BioSciences, Seattle, WA, USA). The following morning cells were washed in 1X PBS as before and then incubated in a fluorescein isothiocyanate conjugated anti-rabbit secondary antibody (Sigma-Aldrich, St. Louis, MO, USA) for 1 h. Cells were washed again and then chambers were removed. Cells were mounted using Fluoroshield with 4’,6-diamidino-2-phenylindole (DAPI, Sigma-Aldrich) and imaged using Cell Sense software (version 1.4, Olympus, Waltham, MA, USA) on an Olympus IX51 microscope (Waltham, MA, USA).

### 4.4. Statistical Analysis

All experiments were independently performed in triplicate at a minimum. All data were expressed as mean ± S.E. Statistical analysis was performed using Analysis of Variance (ANOVA, Bonferoni post hoc) with the statistical software GraphPad Prism 6 (GraphPad Software, La Jolla, CA, USA). The differences were considered statistically significant when *p* < 0.05.
